# Field-collected *Triatoma sordida* from central Brazil display high microbiota diversity that varies with regard to developmental stage and intestinal segmentation

**DOI:** 10.1371/journal.pntd.0006709

**Published:** 2018-08-23

**Authors:** Joana L. Oliveira, Juliano C. Cury, Rodrigo Gurgel-Gonçalves, Ana C. Bahia, Fernando A. Monteiro

**Affiliations:** 1 Laboratório de Epidemiologia e Sistemática Molecular, Instituto Oswaldo Cruz, Fiocruz, Rio de Janeiro, Brazil; 2 Laboratório de Bioquímica de Insetos e Parasitos, Instituto de Biofísica Carlos Chagas Filho, Universidade Federal do Rio de Janeiro, Rio de Janeiro, Brazil; 3 Departamento de Ciências Exatas e Biológicas, Universidade Federal de São João del-Rei, *Campus* de Sete Lagoas, Sete Lagoas, Minas Gerais, Brazil; 4 Laboratório de Parasitologia Médica e Biologia de Vetores, Faculdade de Medicina, Universidade de Brasília, Brasília, Distrito Federal, Brazil; 5 Instituto Nacional de Ciência e Tecnologia em Entomologia Molecular, Rio de Janeiro, Brazil; Liverpool School of Tropical Medicine, UNITED KINGDOM

## Abstract

**Background/Methodology:**

Triatomine bugs are the vectors of *Trypanosoma cruzi*, the agent of Chagas disease. Vector control has for decades relied upon insecticide spraying, but insecticide resistance has recently emerged in several triatomine populations. One alternative strategy to reduce *T*. *cruzi* transmission is paratransgenesis, whereby symbiotic bacteria are genetically engineered to produce *T*. *cruzi*-killing proteins in the vector’s gut. This approach requires in-depth knowledge of the vectors’ natural gut microbiota. Here, we use metagenomics (16S rRNA 454 pyrosequencing) to describe the gut microbiota of field-caught *Triatoma sordida*–likely the most common peridomestic triatomine in Brazil. For large nymphs (4^th^ and 5^th^ stage) and adults, we also studied separately the three main digestive-tract segments–anterior midgut, posterior midgut, and hindgut.

**Principal findings:**

Bacteria of four phyla (12 genera) were present in both nymphs (all five stages) and adults, thus defining *T*. *sordida*’s ‘bacterial core’: Actinobacteria (*Brevibacterium*, *Corynebacterium*, *Dietzia*, *Gordonia*, *Nitriliruptor*, *Nocardia*, *Nocardiopsis*, *Rhodococcus*, and *Williamsia*), Proteobacteria (*Pseudomonas* and *Sphingobium*), and Firmicutes (*Staphylococcus*). We found some clear differences in bacterial composition and relative abundance among development stages; overall, Firmicutes and Proteobacteria increased, but Actinobacteria decreased, through development. Finally, the bacterial microbiotas of the bugs’ anterior midgut, posterior midgut, and hindgut were sharply distinct.

**Conclusions/Significance:**

Our results identify the ‘bacterial core set’ of *T*. *sordida* and reveal important gut microbiota differences among development stages–particularly between 1^st^–3^rd^ stage nymphs and adults. Further, we show that, within any given development stage, the vectors’ gut cannot be regarded as a single homogeneous environment. Cultivable, non-pathogenic ‘core’ bacterial species may now be tested as candidates for paratransgenic control of *T*. *cruzi* transmission by *T*. *sordida*.

## Author summary

Triatomines are blood-sucking bugs that transmit *Trypanosoma cruzi*, the agent of Chagas disease. Insecticide spraying has been very successful at controlling house-infesting bugs, but some triatomines have developed insecticide resistance. One alternative disease-control strategy involves modifying the genomes of bacteria living inside the bugs’ gut so that they produce *T*. *cruzi*-killing substances. An obvious requirement of this strategy is in-depth knowledge of the natural bacterial community (the ‘microbiota’) of the vectors’ gut. In this study, we evaluated bacterial diversity inside the guts of field-collected *Triatoma sordida*–a common pest in parts of Brazil, Argentina, Paraguay, and Bolivia. We found that a ‘core’ set of 12 bacterium genera occur in both immature (five stages) and adult bugs (male and female), but also noticed changes in the gut’s microbiota through development. We finally investigated whether and to what degree the microbiota differed across the bugs’ three intestinal segments, and found clear variation. Our results will help pinpoint suitable candidates for genetic modification aimed at controlling *T*. *cruzi* inside *T*. *sordida*–that is, non-pathogenic bacteria that belong to the ‘core’ set and are easy to rear and maintain in the lab.

## Introduction

Chagas disease is a potentially life-threatening illness caused by the protozoan *Trypanosoma cruzi*. *T*. *cruzi* is a parasite of mammals primarily transmitted through the feces of infected vectors–blood-sucking bugs of the subfamily Triatominae. Chagas disease is the fourth most important infectious disease in the Americas, with about 8 million people infected and at least 120 million people living at risk of contagion [1].

Triatomines are hemimetabolous insects with five immature nymphal stages between egg and adulthood. Adults are sexually mature and have wings, but both nymphs and adults display similar feeding behavior and occupy the same habitats. As all stages feed on vertebrate blood, they are all prone to acquiring and transmitting *T*. *cruzi* [2]. Once the parasite arrives at the triatomine’s midgut with a blood meal, it comes into contact with the local microbiota. To survive and develop inside the insect’s gut, the parasite must evade the immune system and avoid detrimental interactions with the microbiota [3]. In the anterior midgut the parasite differentiates from the blood-borne trypomastigote to a spheromastigote, and then to the epimastigote replicative form. Elongated epimastigotes attach to the waxy cuticle of the hindgut wall, multiply by binary fission, and change into the infective metacyclic trypomastigote form, which is excreted with the feces from the rectum, ready to begin a new infective cycle [4].

Of the 152 formally described triatomine bug species, 67 are known to occur in Brazil [5]; the four species most frequently caught in and around houses in the country are *Panstrongylus megistus*, *Triatoma brasiliensis*, *T*. *pseudomaculata*, and *T*. *sordida* [6,7]. *T*. *sordida* is native to the Cerrado savannahs, although ecological niche modeling suggests that it may also occur in the semiarid Caatinga and the Pantanal floodplains [8,9]. To the south, it has also been recorded in the Chaco of Argentina, Bolivia, and Paraguay, suggesting that the taxon might in fact be a species complex [10–12].

Chagas disease control has for decades relied on the reduction of domestic and peridomestic vector populations through pyrethroid insecticide spraying [13]. In recent years, however, insecticide resistance has been detected in several triatomine populations [14]. This has brought renewed thrust to research aimed at the development of alternative control approaches. For example, both *in vitro* [15] and *in vivo* experiments [16–18] have shown that the gut microbiota of *Rhodnius prolixus* can modulate *T*. *cruzi* survival and development. A more direct attempt at disease control has been paratransgenesis–the use of transgenic gut bacteria that secrete *T*. *cruzi*-killing proteins [19] or express dsRNAs that reduce survival or hinder reproduction of the vectors [20]. The use of the insect microbiota to combat infection and transmission thus represents an interesting alternative to traditional control methods.

Elucidating the role played by the gut microbiota in vector survival and *T*. *cruzi* infection and transmission may thus help devise novel disease-control strategies [21–23]. One key limitation of our current knowledge about the microbiota of triatomine bugs, however, is that little is known regarding field-collected specimens. Additionally, although enzymatic activities and nutrient absorption differ across digestive-tract segments, the gut of triatomines has hitherto been studied whole, as if it were a single homogeneous environment.

Until recently, the investigation of bacterial diversity in insect guts rested upon the isolation and identification of cultivable bacteria–a method that inevitably misses many taxa. DNA sequencing, and in particular high-throughput technologies and metagenomics, now allow fast and accurate detection and determination of bacterial diversity (including non-cultivable species) virtually anywhere–for example, inside animal hosts [24]. In this work, we combined a metagenomics approach with bacterial-community analyses to investigate the gut microbiota of field-collected *T*. *sordida*. We asked whether and how the microbiota changes through bug development, and, for a subset of bugs, determined and compared the segment-specific microbiotas of the anterior midgut, the posterior midgut, and the hindgut.

## Material and methods

### Bug collection

Triatomine bugs were manually captured from chicken coops of six dwellings in a rural area of Posse (14°05’19”S; 46°21’18”W), state of Goiás, Brazil. Property owners provided oral informed consent to have their chicken coops surveyed for triatomines. The region is within the Cerrado biome and has a dry tropical climate with a dry season from May to September, a rainy season from December to March, and two shorter, transitional seasons. Fieldwork took place in three (five-day) trips in December 2013 (rainy season; 201mm total rainfall, 27.8°C mean temperature), May 2014 (dry season; 107mm, 25.2°C), and November 2014 (transitional season; 170mm; 26.9°C). The bugs were transported alive to the laboratory, where they were morphologically identified based on Lent and Wygodzinsky’s keys [8].

### Specimen selection and dissection

We randomly selected five apparently fully blood-engorged bugs of each development stage (1^st^ to 5^th^ instar nymphs plus male and female adults) for dissection. Each stage-specific pool (pyrosequencing sample) included bugs caught in the dry (two bugs), rainy (two bugs), and transitional (one bug) seasons. Prior to dissection, we sterilized each bug’s external cuticle by immersion in 70% ethanol (2 min) followed by five rinses in phosphate-buffered saline (PBS) [25]. Bugs were individually dissected on sterile glass slides with sterilized forceps and disposable needles. After dissection, the guts of larger nymphs (4^th^ and 5^th^ stage) and male and female adults (five specimens each) were cut into three segments corresponding to the major anatomic sections of the bugs’ digestive tract–the anterior midgut (AM), the posterior midgut (PM) and the hindgut (H). Due to their small size, the guts of 1^st^, 2^nd^, and 3^rd^ stage nymphs were left whole. For comparisons of whole guts among development stages, the three segments of 4^th^-5^th^ stage nymphs and adults were analyzed jointly (i.e., grouping the sequences of the three libraries together). Although this grouping might introduce taxonomic biases and thus brings limitations to the analyses, it allows for a more comprehensive view of bacterial-community changes along the entire development process. Comparisons involving whole guts from 1^st^, 2^nd^, and 3^rd^ stages and each of the three intestinal segments from 4^th^-5^th^ stage nymphs and adults are presented as supporting information (S1 Table, S1 and S2 Appendices, all in S1 Text). Dissected material was isolated in 1.5 ml tubes, aseptically macerated in 300 μl of PBS solution with 50% glycerol, and stored at −80°C until DNA extraction.

### Molecular detection of *T*. *cruzi* in *T*. *sordida* samples

To identify the presence of *T*. *cruzi* in field-collected bugs, DNA was extracted from individual triatomines with the Qiamp blood mini kit (Qiagen) to PCR- amplify the kinetoplast DNA of *T*. *cruzi* as described by Cummings et al. [26]. The reaction mix was prepared using a Taq PCR Master Mix Kit (Qiagen; as recommended by the manufacturer), 10 pmol of each primer (TCZ-F* 5'-GCTCTTGCCCACAMGGGTGC-3' and TCZ-R 5'-CCAAGCAGCGGATAGTTCAGG-3' [26], and 10ng of DNA in a final volume of 20 μl. Three μl of the PCR products were run in a 2% agarose-TBE gel stained with ethidium bromide (10 μg/ml); samples yielding a 182-bp band were considered positive for *T*. *cruzi* DNA. DNA extracted from two *T*. *cruzi* strains (CL Brener and Y) was used as a positive control. The Y strain belongs to the major lineage circulating in the study area, *T*. *cruzi* II, whereas CL Brener is a *T*. *cruzi* I/*T*. *cruzi* II hybrid.

### Metagenomic library construction and 454 pyrosequencing

The following assemblages were considered as individual samples for DNA library construction: the whole gut of 1^st^ stage nymphs (1I); the whole gut of 2^nd^ stage nymphs (2I); the whole gut of 3^rd^ stage nymphs (3I); the anterior midgut of 4^th^ stage nymphs (4AM); the posterior midgut of 4^th^ stage nymphs (4PM); the hindgut of 4^th^ stage nymphs (4H); the anterior midgut of 5^th^ stage nymphs (5AM); the posterior midgut of 5^th^ stage nymphs (5PM); the hindgut of 5^th^ stage nymphs (5H); the anterior midgut of adult females (FAM); the posterior midgut of adult females (FPM); the hindgut of adult females (FH); the anterior midgut of adult males (MAM); the posterior midgut of adult males (MPM); and the hindgut of adult males (MH).

Sample codes are composed of a first character that identifies the bugs’ development stage (4, 4^th^ stage nymphs; 5, 5^th^ stage nymphs; F, adult female; M, adult male), followed by letters that identify intestinal segments (AM, anterior midgut; PM, posterior midgut; H, hindgut)

We extracted DNA with the DNeasy Blood & Tissue Kit (Qiagen) according to the manufacturer’s instructions. The hypervariable regions (V3 to V5) of the bacterial 16S rRNA gene were amplified with primers 357F (5’-CCTACGGGAGGCAGCAG-3’) and 926R (5’-CCGTCAATTCMTTTRAGT-3’) containing 454 sequencing adapters and Multiplex Identifier (MID) tags [27]. PCR was performed with High Fidelity Platinum Taq DNA Polymerase (Invitrogen), with initial denaturation at 95°C for 2 min and 30 cycles of denaturation at 95°C for 20 sec, annealing at 50°C for 30 sec, and extension at 72°C for 5 min. Each 16S rRNA amplicon library was constructed from five independent PCRs pooled in equimolar concentration. PCR products were purified with the Agencourt AMPure XP kit (Beckman Coulter). Pyrosequencing was performed using a 454 Genome Sequencer Junior System (Roche).

### Bacterial 16S rRNA assembly and taxonomic classification

We removed low-quality sequences shorter than 250 nucleotides or containing more than one ambiguous base, as well as sequences of the 16S rRNA primers and MID tags, using the trim.seqs script of Mothur v.1.30.2 [28]. The remaining sequences were aligned against the SILVA alignment database (http://www.mothur.org/w/images/9/98/Silva.bacteria.zip). We used Mothur pre.cluster scripts denoise sequences, and the screen.seq, filter.seq, and chimera.slayer scripts to screen for high-quality sequences. Then we used Mothur sub.sample scripts to (i) assemble three normalized subsets of sequences from 4^th^-5^th^ nymphal stages and adults and (ii) merging their respective anterior midgut, posterior midgut and hindgut libraries. Operational taxonomic units (OTUs) were determined using the cluster script with the nearest-neighbor algorithm and a 3% distance level cutoff (see [28,29]). We classified bacteria based on each sequence’s best match in the SILVA database. Sequences identified as DNA from mitochondria, Archaea, and Eukarya, as well as singletons, were removed from the bacterial community analysis.

### Operational Taxonomic Unit (OTU)-based approach for bacterial community analysis

We used Good’s coverage index (the number of OTUs sampled more than once divided by the total number of OTUs), as implemented in Mothur, to estimate sequencing depths [25]. Rarefaction curves were produced by plotting the number of unique sequence tags as a function of the number of randomly sampled tags with the vegan package in the R computing environment [30,31].

We computed OTU richness as the number of observed OTUs; however, to ensure that our richness estimate was reliable we used the bias-corrected Chao1 estimator. We also computed Shannon’s diversity index, which takes into account both the abundance and the evenness of species in a community [32]. These indices were calculated with the Mothur software [28].

In this paper, we define *T*. *sordida*’s ‘bacterial core’ as the set of bacterial OTUs that are present in all of the bug’s development stages–that is, the intersection of all development stage-specific OTU sets.

### Statistical comparison of *T*. *sordida* bacterial communities

Nonmetric multidimensional scaling (NMDS) represents the pairwise dissimilarity between samples in a low-dimensional space [33]. We used the ‘ordinate’ function of the R Phyloseq package [34] to simultaneously perform weighted UniFrac and a Principal Coordinates Analysis (PCoA) using differences in OTU relative abundances within each sample. We conducted exploratory analyses of similarities (ANOSIM), with Bonferroni-adjusted p-values, to assess and compare the differences between the groups identified through PCoA [33].

## Results

### *T*. *sordida* collection and *T*. *cruzi* infection

We collected 304 *T*. *sordida* specimens; our kDNA PCR did not detect *T*. *cruzi* DNA in any of the samples (see S2 Table in S1 Text, for details).

### Bacterial diversity of the *T*. *sordida* intestinal microbiota

Pyrosequencing of 15 *T*. *sordida* samples (i.e., 15 pools of five whole guts or five gut segments) generated a total of 98,872 good-quality sequences (overall abundance ≥ 1%; mean±SE 6591.5±1072.1 sequences per sample). These sequences were taxonomically identified to the genus level based on a 97% sequence similarity cutoff. Sequences were clustered into 52 bacterial OTUs representing 49 genera in 38 families and four phyla. Rarefaction curves, supported by Good’s coverage index, showed that sampling depth was sufficient (>0.93 mean±SE 0.959±0.003) to accurately characterize *T*. *sordida*’s bacterial communities (S3 Table and S3 Appendix in S1 Text).

### Bacterial diversity across *T*. *sordida* development stages

This subsection addresses the question, “does *T*. *sordida*’s gut bacterial community change through bug development?” The Chao1 index of OTU richness increased from 25.0±2.30 SE OTUs in 1^st^ stage nymphs to 50.0±2.10 SE OTUs in both 5^th^ stage nymphs and adult males. Similarly, Shannon’s diversity index rose from 1.44±1.05 SE bits in 1^st^ stage nymphs to 3.37±1.20 SE bits in adult males (Table 1).

**Table 1:** Bacterial richness and diversity in *Triatoma sordida*’s gut microbiota through bug development. Bacterial Operational Taxonomic Units (OTUs) were defined based on a 97% 16S rRNA sequence identity cutoff.

Sample*	N^o^. observed OTUs	Chao1 richness estimator (mean±SE OTUs)	Shannon’s diversity index (mean±SE bits)
1I	23	25.0 ± 2.30	1.44 ± 1.05
2I	41	42.2 ± 1.20	2.77 ± 1.12
3I	36	37.0 ± 1.25	2.94 ± 1.10
4I	51	49.0 ± 1.50	3.18 ± 2.10
5I	50	50.0 ± 2.10	3.35 ± 1.25
FI	51	48.0 ± 3.00	3.26 ± 1.20
MI	51	50.0 ± 2.10	3.37 ± 1.20

*Samples: 1I–3I, whole intestine of 1^st^ to 3^rd^ stage nymphs; 4I to MI, pooled results from separately processes samples from the anterior midgut, posterior midgut, and hindgut of 4^th^ and 5^th^ stage nymphs (4I and 5I, respectively) and adult female and male bugs (FI and MI, respectively)

**Fig 1:**
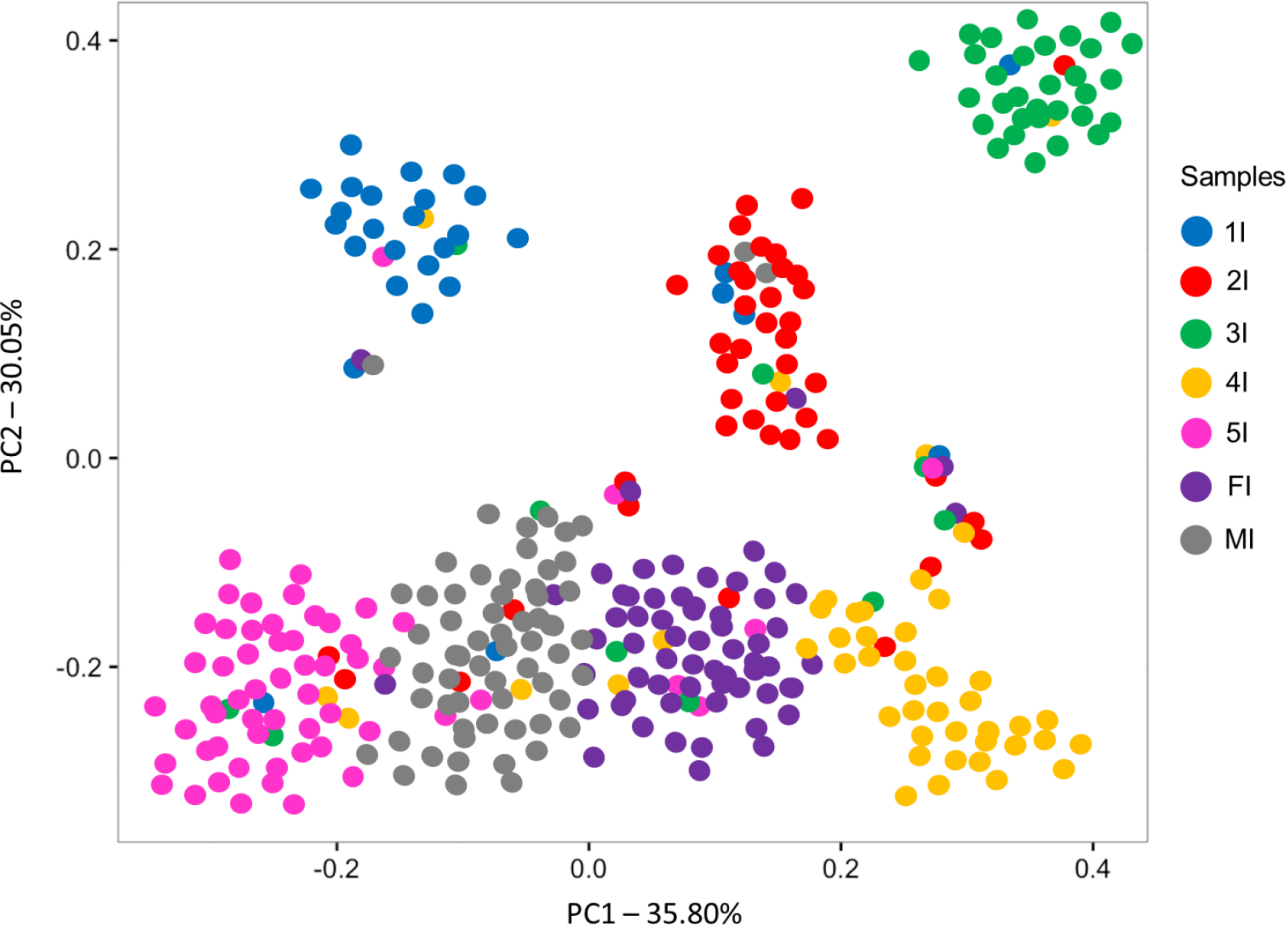
Principal Coordinate Analysis (PCoA) of weighted UniFrac distances comparing *Triatoma sordida*’s gut bacterial communities through development. Weighted UniFrac distance matrices were calculated for each sample using one representative sequence for each OTU (3%) with Bonferroni correction. Axes represent the greatest proportion of variance in the communities for each comparison. Samples: 1I–3I, whole intestine of 1^st^ to 3^rd^ stage nymphs; 4I to MI, pooled results from separately processes samples from the anterior midgut, posterior midgut, and hindgut of 4^th^ and 5^th^ stage nymphs (4I and 5I, respectively) and adult female and male bugs (FI and MI, respectively).

A representative sequence of each OTU present in each sample was used in a principal coordinate analysis (PCoA) based on weighted UniFrac distances as well as in ANOSIM. Principal Coordinate Analysis (PCoA) revealed a trend towards separation of 1^st^–3^rd^ stage nymphs from older nymph stages and adults along Axis 2 (Fig 1). ANOSIM results confirmed OTU divergence between adults and the first three nymphal stages; they suggested, in addition, that the microbiota of 4^th^ stage nymphs differed from that of adult males but not from that of adult females (Table 2).

**Table 2:** Analysis of similarity (ANOSIM) of *Triatoma sordida* gut bacterial communities through development: p values. Statistically significant differences (p < 0.008 after Bonferroni correction) are shown in bold.

Samples*	1I	2I	3I	4I	5I	FI	MI
1I	-	0.0682	0.0173	0.0233	0.0471	**0.0001**	**0.0001**
2I		-	0.0122	0.0111	0.0411	**0.0001**	**0.0007**
3I			-	0.8622	0.0233	**0.0006**	**0.0003**
4I				-	0.0623	0.1314	**0.0007**
5I					-	0.0097	0.5204
FI						-	0.6422
MI							-

*Samples: 1I–3I, whole intestine of 1^st^ to 3^rd^ stage nymphs; 4I to MI, pooled results from separately processed samples from the anterior midgut, posterior midgut, and hindgut of 4^th^ and 5^th^ stage nymphs (4I and 5I, respectively) and adult female and male bugs (FI and MI, respectively)

**Fig 2:**
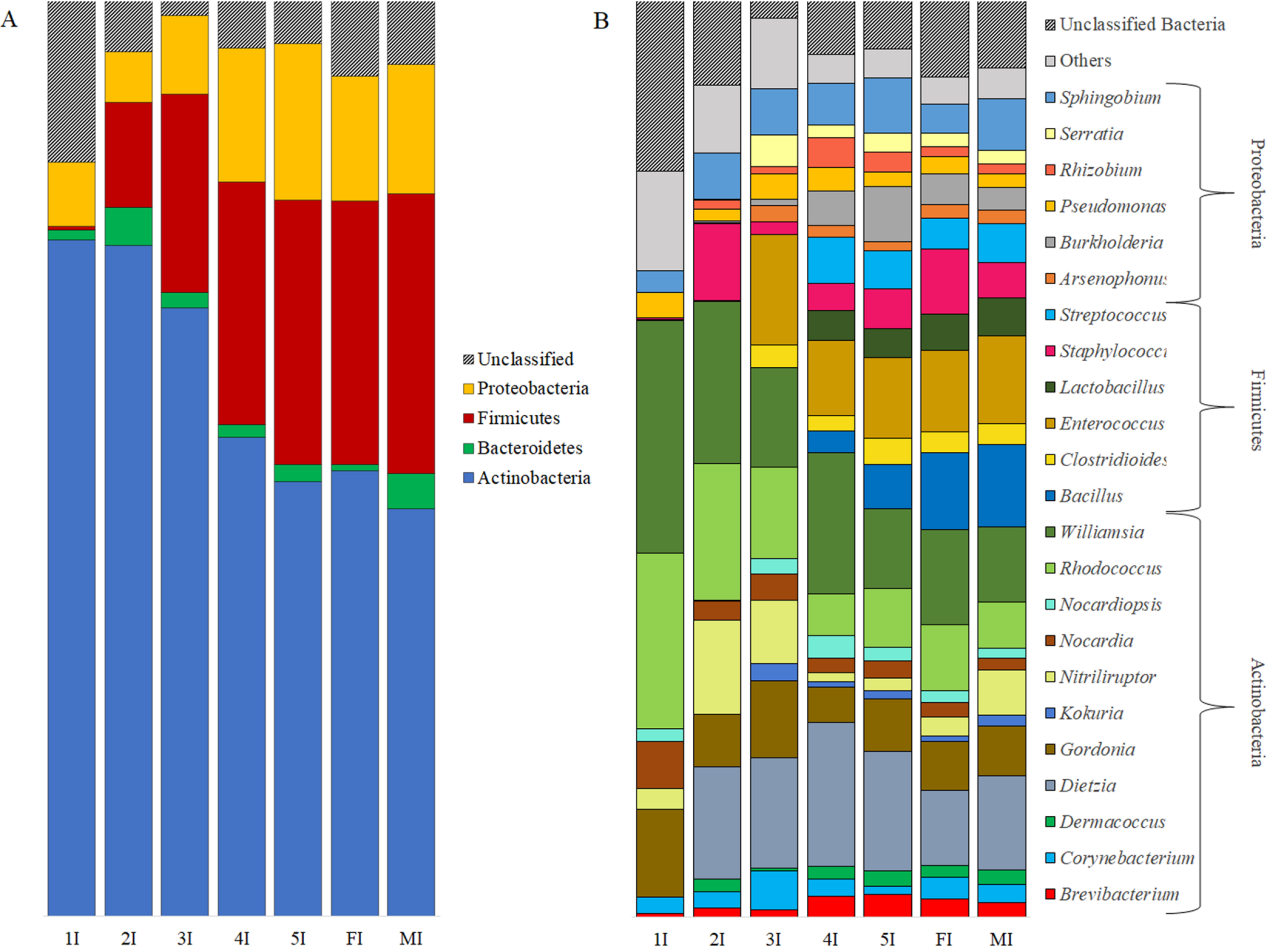
**Relative abundance of gut bacterial phyla (A) and genera (B) through *Triatoma sordida* development.** Samples: 1I–3I, whole intestine of 1^st^ to 3^rd^ stage nymphs; 4I to MI, pooled results from separately processes samples from the anterior midgut, posterior midgut, and hindgut of 4^th^ and 5^th^ stage nymphs (4I and 5I, respectively) and adult female and male bugs (FI and MI, respectively).

Taxonomic classification of *T*. *sordida*’s gut microbiota revealed the steady presence of four bacterial phyla (Actinobacteria, Bacteroidetes, Firmicutes, and Proteobacteria) in all of the bugs’ development stages. Actinobacteria was the predominant phylum (Fig 2A), particularly in the first three nymph stages. The second most abundant bacterial phylum was Firmicutes, which was present in very low numbers in 1^st^ stage nymphs (0.34%) but increased sharply in abundance in 2^nd^ stage nymphs and remained high until adulthood. There was an apparent increase of Firmicutes and Proteobacteria, at the expense of Actinobacteria, with bug development (Fig 2A).

We found no signs of genus-level bacterial dominance or abrupt change in relative abundance with bug development (Fig 2B). Twelve bacterial genera were common to all development stages (*Brevibacterium*, *Corynebacterium*, *Dietzia*, *Gordonia*, *Nitriliruptor*, *Nocardia*, *Nocardiopsis*, *Rhodococcus*, *Pseudomonas*, *Sphingobium*, *Staphylococcus*, and *Williamsia*), with no obvious differences in abundance. No single development stage had unique bacterium genera (Fig 2B). The most abundant genera in the 1^st^ and 2^nd^ stages were *Williamsia* (22.37% and 17.69%, respectively) and *Rhodococcus* (16.85% and 14.91%, respectively) (Fig 2B). *Dietzia* and *Enterococcus* were the most abundant genera in 3^rd^ stage nymphs (12.98% and 12.16%, respectively). *Clostridioides*, *Enterococcus*, *Kocuria*, and *Serratia* were present from the 3^rd^ stage onwards (Fig 2B). Species of *Bacillus*, *Streptococcus*, and *Lactobacillus* appeared in 4^th^ stage nymphs and remained until adulthood (Fig 2B).

### Bacterial diversity in *T*. *sordida* intestinal segments I: Variation across segments

This subsection addresses the question, “does *T*. *sordida*’s gut microbiota composition differ across the three intestinal segments for each development stage?” Estimates of bacterial OTU richness and diversity in each intestinal segment and are shown in Table 3. Overall, we found no clear differences in bacterial OTU diversity across intestinal segments in the development stages we studied (Table 3).

**Table 3:** Bacterial taxon richness and diversity in *Triatoma sordida* gut. Operational Taxonomic Units (OTUs) were defined based on a 97% 16S rRNA sequence identity cutoff.

Sample*	No. observed OTUs	Chao1 richness estimator (mean±SE OTUs)	Shannon’s diversity index (mean±SE bits)
4AM	36	32.5 ± 0.70	2.89 ± 0.75
4PM	44	44.2 ± 0.35	3.20 ± 0.25
4H	33	28.5 ± 1.06	2.81 ± 0.80
5AM	44	41.7 ± 1.06	3.11 ± 1.10
5PM	38	38.5 ± 0.70	2.93 ± 0.90
5H	42	41.5 ± 0.70	2.92 ± 0.90
FAM	43	41.0 ± 1.41	2.85 ± 0.38
FPM	41	41.5 ± 0.70	2.82 ± 0.72
FH	44	43.5 ± 0.70	2.91 ± 0.90
MAM	48	46.0 ± 1.41	3.17 ± 1.20
MPM	42	39.0 ± 1.41	2.90 ± 0.90
MH	43	41.5 ± 0.70	2.95 ± 1.25

* Sample codes are composed of a first character that identifies the bugs’ development stage (4, 4^th^ stage nymphs; 5, 5^th^ stage nymphs; F, adult female; M, adult male), followed by letters that identify intestinal segments (AM, anterior midgut; PM, posterior midgut; H, hindgut)

**Fig 3:**
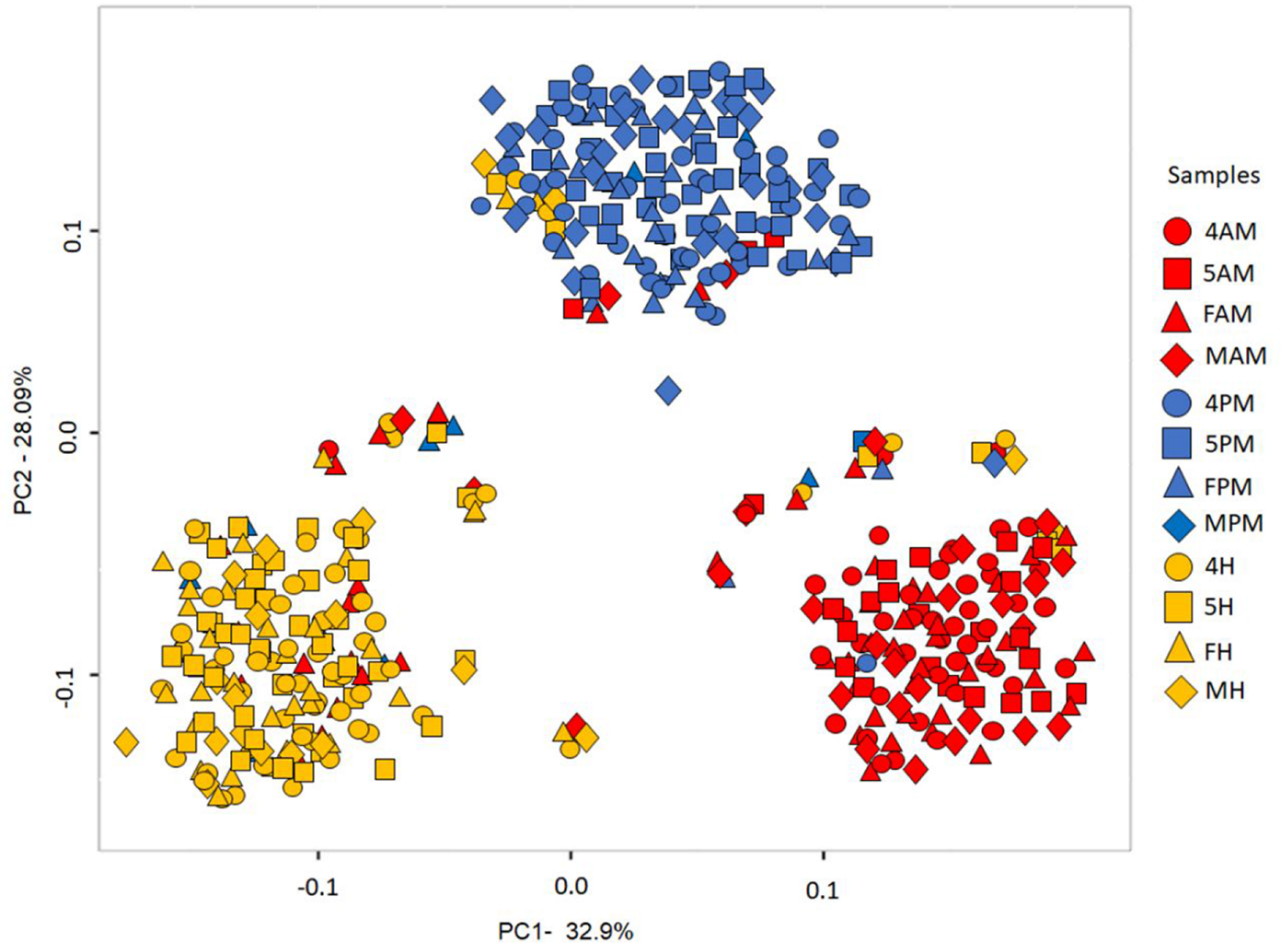
Principal Coordinate Analysis (PCoA) of weighted UniFrac distances comparing *Triatoma sordida*’s gut bacterial communities across intestinal segments. Sample codes are composed of a first character that identifies the bugs’ development stage (4, 4^th^ stage nymphs; 5, 5^th^ stage nymphs; F, adult female; M, adult male), followed by letters that identify intestinal segments (AM, anterior midgut; PM, posterior midgut; H, hindgut).

Bacterial communities present in the intestinal segments were also compared using PCoA based on pairwise weighted UniFrac distances and ANOSIM. PCoA-based comparisons revealed differences in the intestinal segment-specific gut microbiota of each development stage (4^th^ and 5^th^ stages nymphs and adults) (Fig 3; S4 and S5 Appendices and S5 Table, all in S1 Text). ANOSIM results suggested that (i) all three intestinal segments of 4^th^ stage nymphs have distinct microbiotas; (ii) in 5^th^ stage nymphs, the hindgut microbiota is different from that in the other two segments; and (iii) in adults, the posterior midgut microbiota may be slightly different from that in the other two segments (Table 4).

**Table 4:** Analysis of similarity (ANOSIM) of *Triatoma sordida* gut bacterial communities across intestinal segments: p values. Statistically significant differences (p < 0.01 after Bonferroni correction) are shown in bold; those involving biologically meaningful comparisons are italicized (i.e. between same stage segments and between same segments through development.

Sample*	4AM	4PM	4H	5AM	5PM	5H	FAM	FPM	FH	MAM	MPM	MH
4AM	-	***0*.*0001***	***0*.*0052***	0.0826	**0.0008**	**0.0051**	0.0155	**0.0015**	**0.0023**	0.0457	**0.0013**	**0.0001**
4PM		*-*	***0*.*0008***	0.3495	0.2010	**0.0012**	**0.0001**	0.0729	0.2733	0.0826	0.0802	**0.0022**
4H			-	**0.0094**	0.0280	0.0495	**0.0070**	0.2395	***0*.*0010***	**0.0085**	0.0999	0.0981
5AM				-	0.0290	***0*.*0039***	0.0222	**0.0052**	**0.0090**	***0*.*0012***	0.0119	**0.0099**
5PM					-	***0*.*0013***	0.0224	0.0829	**0.0042**	0.0822	0.0830	0.5115
5H						-	**0.0053**	0.0334	0.0110	0.1000	0.2113	0.0612
FAM							-	***0*.*0015***	0.0110	***0*.*0015***	**0.0010**	0.1002
FPM								-	**0.0083**	0.0322	0.0210	**0.0011**
FH									-	0.0112	0.3924	0.1001
MAM										-	0.0100	0.1021
MPM											-	***0*.*0010***
MH												-

* Sample codes are composed of a first character that identifies the bugs’ development stage (4, 4^th^ stage nymphs; 5, 5^th^ stage nymphs; F, adult female; M, adult male), followed by letters that identify intestinal segments (AM, anterior midgut; PM, posterior midgut; H, hindgut)

**Fig 4:**
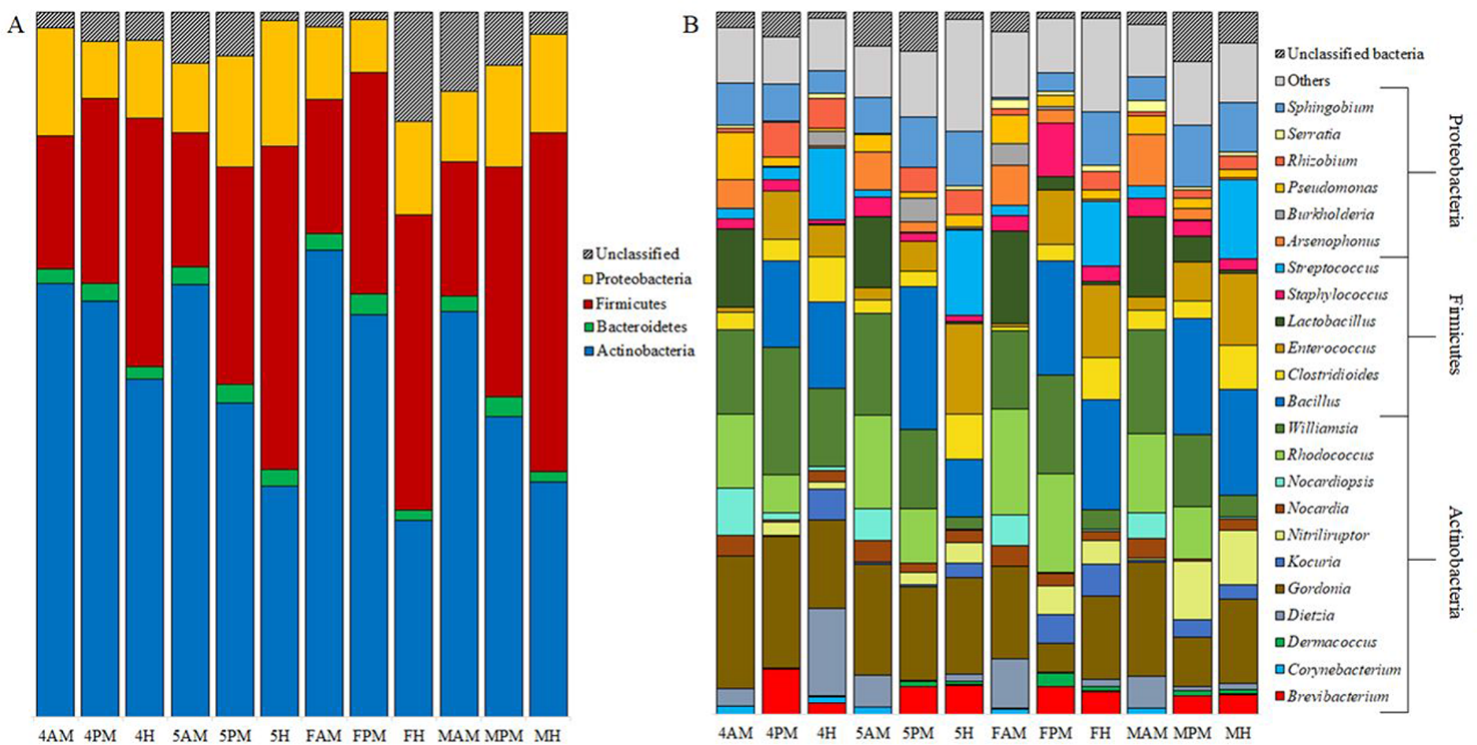
**Relative abundance of gut bacterial phyla (A) and genera (B) across *Triatoma sordida* intestinal segments.** Sample codes are composed of a first character that identifies the bugs’ development stage (4, 4^th^ stage nymphs; 5, 5^th^ stage nymphs; F, adult female; M, adult male), followed by letters that identify intestinal segments (AM, anterior midgut; PM, posterior midgut; H, hindgut).

We found a consistent increase in abundance of Firmicutes, at the expense of Actinobacteria, from anterior midgut to hindgut in all development stages (Fig 4A). This was also the case when samples were clustered together by intestinal segment (S6A Appendix in S1 Text).

The relative abundance of some bacterial genera tended to change across intestinal segments (Fig 4B). For example, *Enterococcus* was rare in the anterior midgut (4AM 0.71%; 5AM 1.69%; FAM 0.41%; and MAM 1.49%) but became more common in the posterior midgut (4PM 6.85%; 5PM 4.31%; FPM 7.70%; and MPM 5.48%) and, except for 4^th^ stage nymphs, in the hindgut (4H 4.58%; 5H 12.87%; FH 10.36%; and MH 10.37%) (Fig 4B). *Kocuria* is another example–it was nearly absent from the anterior and posterior midguts of 4^th^ (0.04% and 0.25%, respectively) and 5^th^ stage nymphs (0.07% and 0.39%, respectively), but rose to 4.38% and 2.13% in the hindguts (Fig 4B). *Kocuria* was likewise nearly absent from the adult bugs’ anterior midgut (females 0.01%, males 0.02%), but its abundance was much higher, particularly in females, in the posterior midgut (4.47% and 2.48%) and the hindgut (4.40% and 2.08%) (Fig 4B).

To further investigate these differences in the microbiota of the three intestinal segments, we pooled the sequences from each stage-specific sample by intestinal segment (S4 and S5 Tables and S4 Appendix, all in S1 Text). PCoA analysis of these intestinal segment-specific sequence pools confirmed the distinctness of the three intestinal segments, and ANOSIM suggested that the hindgut was significantly different from the two other segments (S5 Table and S4 Appendix in S1 Text).

### Bacterial diversity in *T*. *sordida* intestinal segments II: Variation through development

This subsection addresses the question, “does *T*. *sordida*’s gut microbiota composition in each intestinal segment change with development (i.e. from one stage to the next)?” Estimates of bacterial OTU richness and diversity in each intestinal segment through development are shown in Table 3. PCoA and ANOSIM revealed some indication of within-segment homogeneity (Fig 3). The only differences suggested by ANOSIM were (i) between the anterior midgut microbiota of males and that of 5^th^ stage nymphs and females; and (ii) between the hindguts of 4^th^ stage nymphs and females (Table 4).

We found no differences in bacterial phylum composition in the anterior midgut across development stages. The posterior midgut microbiota was similar in phylum composition both in 4^th^ stage nymphs and females and in 5^th^ stage nymphs and males. A decrease in the abundance of Actinobacteria was observed in the hindgut of 4^th^ to 5^th^ stage nymphs and adults (Fig 4B).

We identified only small differences in bacterial dominance or abundance when we compared intestinal segment-specific microbiotas through development. Thus, in the posterior midgut, *Gordonia* decreased in abundance from the 4^th^ nymphal stage (18.68%) to females (4.15%); *Nitriliruptor*, on the other hand, increased in abundance from the 5^th^ nymphal stage (1.74%) to adults (4.07% for females and 8.27% for males). In the hindgut, *Dietzia* decreased in abundance from the 4^th^ nymphal stage (12.4%) to males (1.14%), and *Williamsia* also decreases in abundance from the 4^th^ nymphal stage (11.18%) to the 5^th^ stage (1.79%) and adults (2.7%) (Fig 4B).

The bugs’ anterior midguts had a higher abundance of *Williamsia*, *Rhodococcus*, *Lactobacillus*, and *Nocardia* (16.41%, 11.81%, 10.78%, and 4.52%, respectively). *Bacillus* (17.10%) was the most abundant genus in posterior midgut samples, followed by *Williamsia* and *Gordonia* (15.13% and 10.86%). *Bacillus* was also dominant in hindgut samples (13.65%), followed by *Gordonia* and *Enterococcus* (12.75% and 9.34%, respectively) (Fig 4B, S6B Appendix in S1 Text). Some bacterial genera, although present in all intestinal segments, seemed to favor one specific segment (e.g. *Enterococcus*, *Clostridioides* and *Dermacoccus* are more abundant in the hindgut), whereas other genera were absent from certain segments (e.g. *Bacillus* from the anterior midgut; *Corynebacterium* and *Serratia* from the posterior midgut, and *Rhodococcus* from hindgut samples) (Fig 4B, S6B Appendix in S1 Text).

## Discussion

The three most salient findings of this study are the observations that: (1) the bacterial community of *T*. *sordida*’s intestinal tract undergoes important compositional changes during bug development, with particularly prominent differences between young nymphs (1^st^–3^rd^ stages) and adults; (2) within three development stages tested (4^th^ and 5^th^ stage nymphs plus male and female adults), the three major gut segments contain distinct microbiotas and, hence, cannot be regarded as a single homogeneous environment; and (3) the microbiota of each gut segment does not undergo substantial changes from 4^th^ stage nymphs to adults.

Bacteria found inside insects’ guts may develop many kinds of relations with their hosts, ranging from lethal pathogenesis to obligate mutualism. Some are even unable to stably colonize the gut and are just transient residents quickly eliminated through defecation. In general, gut-colonizing bacteria need to multiply in the gut at a rate that equals or exceeds their rate of elimination [35]. Those that live in stable, close association with the digestive tract of their insect hosts are usually involved in key processes such as resistance against pathogens [36], nutrient supplementation [37,38], digestion [39], or detoxification [40]. In the absence of their gut microbiotas, the mosquito vectors *Aedes aegypti* and *Anopheles gambiae* cannot develop normally [41]. The absence of the symbiotic bacterium *Rhodococcus rhodnii* in *R*. *prolixus*’ gut may result in delayed larval development, defective blood digestion and excretion, underdevelopment of the tracheal system, and, more generally, higher bug mortality [37]. Experimental removal of gut bacteria impairs *Anopheles gambiae*’s immune system and hence affects the interactions between the mosquito and mosquito-borne pathogens of the genus *Plasmodium* [42]. Similarly, symbiotic bacteria of the genus *Wigglesworthia* are key to the normal functioning of the immune system in the tsetse fly, *Glossina morsitans* [43]. Given the important role it plays in insect physiology and, crucially, given its potential for modulating the interactions between disease vectors and the pathogens they transmit, the insect gut microbiota holds much promise for the development of novel vector-borne disease control strategies [22,44].

The gut microbiota of triatomine bugs has been studied using isolation of cultivable bacteria, denaturing gradient gel electrophoresis, and 16S rRNA gene fragments–both by conventional PCR and by next-generation sequencing [45–48]. Although informative, most of these studies relied on laboratory-reared bugs; the extent to which their results may apply to natural populations therefore remains unclear. Our study, in which only field-caught bugs were used, was designed to overcome this common drawback. In addition, we sampled bugs in the dry, rainy, and transitional seasons, thus covering the likely seasonal variation of bacterial communities [49].

All our 304 field-caught *T*. *sordida* tested negative for *T*. *cruzi* kDNA. This agrees with previous reports suggesting that *T*. *cruzi* infections can be rare in peridomestic *T*. *sordida* populations (which, as in our case, are often associated with avian hosts that are refractory to *T*. *cruzi* infection) [50]. Most importantly, infection with *T*. *cruzi* can affect the bugs’ gut microbiota [47]; its absence discounts one major complicating factor and thus allows for a more straightforward interpretation of our results.

### Bacterial diversity of the *T*. *sordida* gut microbiota

We identified 52 bacterial OTUs in the 15 *T*. *sordida* samples we analyzed. This observed OTU richness is higher than reported for field-caught *T*. *pseudomaculata* (23 OTUs) or *T*. *brasiliensis* (35 OTUs) [46]. The numbers of OTUs found in the guts of these *Triatoma* species are, however, much smaller than those reported for other insects–e.g., 300 OTUs in the Asian longhorn beetle *Anoplophora glabripennis* [51] or 417 OTUs in the tiger mosquito *Aedes albopictus* [52]. This suggests that the digestive tract microbiota of triatomines may encompass much fewer phylotypes than the gut microbiotas of other insects. *T*. *sordida* gut-associated OTUs belonged in four main phyla: Actinobacteria, Proteobacteria, Firmicutes, and Bacteroidetes. Species of 12 genera were present in all development stages and both sexes, and can therefore be collectively regarded as *T*. *sordida*’s gut ‘bacterial core set’: *Brevibacterium*, *Corynebacterium*, *Dietzia*, *Gordonia*, *Nitriliruptor*, *Nocardia*, *Nocardiopsis*, *Rhodococcus*, *Williamsia* (Actinobacteria), *Pseudomonas*, *Sphingobium* (Proteobacteria), and *Staphylococcus* (Firmicutes). Given their persistence across all development stages, one should expect that at least some species of these 12 genera play important roles in *T*. *sordida* physiology, so that bugs carrying them in their guts have improved chances of survival and reproduction.

Actinobacteria species of the genus *Rhodococcus* play important roles in the life cycle of triatomine bugs, providing their hosts with essential B-complex vitamins [37]. *Dietzia*, *Gordonia*, and *Williamsia* species, among others, produce secondary metabolites with antibacterial and antifungal properties and that contribute to gut microbiota homeostasis [53,54].

Proteobacteria can modulate the interaction between insect vectors and the pathogens they transmit. Thus, *Pseudomonas putida* decreases *Plasmodium falciparum* levels in *Anopheles gambiae* mosquitoes [23], and *Pseudomonas fluorescens* causes *T*. *cruzi* lysis *in vitro* [55]. It is possible that some of the *Pseudomonas* species present in all *T*. *sordida* development stages and intestinal segments confer some degree of refractoriness to infection by *T*. *cruzi*.

### Bacterial diversity across *T*. *sordida* development stages

The number of phylotypes in the gut bacterial community of *T*. *sordida* increased through bug development, with clear differences in richness between the first nymph stages and later stages (Table 1). We however found no differences in Shannon’s diversity index values among the bugs’ development stages (Table 1).

PCoA analyses revealed a tendency for separation of the first three nymph-stage sequences from those of the later stages (Fig 1), and ANOSIM suggested significant differences in gut bacterial composition between the first three stages and adults, and between 4^th^ stage nymphs and males. These observations strongly suggest that the composition of *T*. *sordida* gut bacterial community changes through bug development. Gut bacterial communities also change through development in other insect groups, such as mosquitoes (e.g., [41]). Mosquitoes, however, undergo complete metamorphosis, with larvae developing in aquatic environments and only adult females feeding on vertebrate blood. In *T*. *sordida*, as in all Hemiptera, metamorphosis is incomplete and morphology, behavior, habitats, and habits including blood-feeding are all similar through development–yet the bugs’ gut microbiota still changes from early to late development stages. We note, in addition, that such changes were observed in bugs caught in chicken coops and, hence, most likely fed on chickens. These observations might indicate that environmental factors including bloodmeal sources have relatively little effect on the bugs’ gut bacterial communities, which may instead be to some degree directly linked to development–through, e.g., maturation of the immune system or selection of particular bacterial species that increase host fitness.

Bacteria of four phyla were present in all development stages: Actinobacteria (the most abundant), Bacteroidetes, Firmicutes, and Proteobacteria (Fig 2A). The abundance of Actinobacteria decreased, whereas Firmicutes and Proteobacteria species became more abundant, through the bugs’ development. These results are consistent with findings of Actinobacteria as the main phylum in field-caught *T*. *brasiliensis*, *T*. *pseudomaculata*, and *R*. *pallescens*; Proteobacteria species, however, predominated in peridomestic *T*. *maculata* [46,48]. Recent studies spanning a more comprehensive taxonomic sample of insects reported a clear dominance of Proteobacteria (60%) and Firmicutes (20%) among 218 and 62 insect species, respectively [56,57]. Proteobacteria species also seem to predominate in mosquitoes [52]. Proteobacteria are commonly found in the gut communities of a wide range of animals, including humans and insects, and are involved in vitamin biosynthesis [54], protection against pathogens [17], and degradation of plant compounds [58]. Proteobacteria species also increased in abundance with the reduction of Actinobacteria species, except for 4^th^ stage nymphs. No single developmental stage presented unique genera.

Although nearly absent from the guts of 1^st^ stage nymphs, Firmicutes rose sharply from the 2^nd^ stage onwards; overall, this was the second most abundant bacterial phylum in our samples. It is worth noting that some genera, such as *Streptococcus* and *Bacillus*, were absent from the first three stages of development, but appeared and became abundant from the 4^th^ stage on; *Lactobacillus* species also appeared only on 4^th^ stage nymphs and remained until adulthood (Fig 2B).

No overall differences were detected between males and females, but we found some evidence of intestinal segment-specific sex differences (see below).

### Bacterial diversity in *T*. *sordida* intestinal segments I: Variation across segments

Our results suggest that the digestive tract of *T*. *sordida* should not be regarded as a single homogeneous environment. Pairwise comparisons of the three intestinal segments of 4^th^ and 5^th^ stage nymphs and adults show at least two significant differences in bacterial community OTU richness per stage (Table 4). One such difference is between the anterior midgut of males and females (note that no differentiation was detected when pooling the segment-specific data). This indicates that unless intestinal segments are analyzed individually, some differences might go undetected.

We observed no differences in OTU diversity among intestinal segments. OTU relative abundance, however, varied significantly among intestinal segments. We found an increase of Firmicutes abundance at the expense of Actinobacteria along the *T*. *sordida* intestinal tract. This was also the case when samples were clustered together by intestinal segment. Certain genera appeared to be better represented in specific segments; for example, *Enterococcus*, *Clostridioides* and *Dermacoccus* were more abundant in the hindgut. On the other hand, some genera were absent from certain segments; for example, *Bacillus* was absent from the anterior midgut, *Corynebacterium* and *Serratia* were absent from the posterior midgut, and *Rhodococcus* was absent from hindgut samples (Fig 4B, S6B Appendix in S1 Text). *Enterococcus* bacteria produce cytolysin, a lytic molecule with activity against diverse prokaryotic and eukaryotic cells such as Gram-positive bacteria, erythrocytes, leucocytes, and epithelial cells [59]. *Serratia marcescens* also secretes cytolysin [60] and is capable of inhibiting *T*. *cruzi* development inside the triatomine gut by attaching itself to the parasite surface [21]. The presence of *Serratia* sp. in the anterior midgut of *T*. *sordida* can contribute to the observed reduction of *T*. *cruzi* numbers in this compartment in the first days of infection [59,60]. Conversely, the absence of *Serratia* sp. in the posterior midgut may facilitate the replication and establishment of *T*. *cruzi* in the bugs’ gut, although the presence of *Enterococcus* may inhibit parasite differentiation in the hindgut.

### Bacterial diversity in *T*. *sordida* intestinal segments II: Variation through development

When each intestinal segment is compared across development stages, there is an indication of within-segment homogeneity in OTU richness. Only minor differences were observed between the anterior midgut of males and 5^th^ stage nymphs and females, and hindguts of 4^th^ stage nymphs and females. No single segment had a notably higher richness among the stages we analyzed. We found no differences in bacterial phylum composition in the anterior midgut across development stages (Fig 4A). In the posterior midgut, phylum composition was very similar in 4^th^ stage nymphs and females, as well as in 5^th^ stage nymphs and males (Fig 4A). The abundance of hindgut Actinobacteria decreased from 4^th^ to 5^th^ stage nymphs to adults (Fig 4A).

These differences in bacterial composition along the three major anatomical segments of *T*. *sordida*’s gut may be explained by the specific function of each segment during blood meal digestion. The anterior midgut of triatomines has a neutral-basic pH near 7.2 and functions as a reservoir of the ingested blood, which remains essentially undigested [61]. Only water elimination, erythrocyte lysis [59], and inhibition of blood clotting by anticoagulants [62] take place. The anterior midgut harbors several symbiotic bacteria [37], which (especially actinomycetes) may reach densities of up to 10^9^ colony-forming units per insect after a blood meal [63]. Actinobacteria species are also predominant in *T*. *sordida*’s anterior midgut (Fig 4A). This dense bacterial population may result from high nutrient contents; in addition, triatomines decrease reactive oxygen species (ROS) levels immediately after blood meal ingestion by reducing the production of mitochondrial superoxide [64]. In contrast, the proliferation of bacteria in the anterior midgut activates the bug’s immune response, as evidenced by the high antibacterial activity seen in this segment when compared to the posterior midgut in *R*. *prolixus* [65]. This immune activation, coupled with the presence of *Serratia* sp. (mentioned above), can be important for the reduction of trypomastigote populations observed in this segment [62,63]. On the other hand, the presence of *T*. *cruzi* also decreases bacterial abundance in the anterior midgut of *R*. *prolixus* in the first days of infection [18]. The parasite can induce a Kazal-type protease inhibitor during the first hours of infection, which allows microbiota modulation and thus its successful maintenance in the host [66].

The posterior midgut is where complete blood digestion and nutrient absorption takes place, with participation of cathepsin L, carboxypeptidases, and aminopeptidases [67]. Symbiont population density is strongly reduced in this intestinal segment after a blood meal [63]. This suggests that proteases involved in blood digestion may also participate in microbiota control. In *R*. *prolixus*, digestion seems to have unequal lytic effects on different *T*. *cruzi* strains [68].

The hindgut receives and stocks blood remains until defecation. We observed a tendency of increasing abundance of Firmicutes species in the hindgut and in the posterior midgut compared with the anterior midgut in all development stages (with the exception of adult males). Firmicutes bacteria also produce antimicrobial molecules such as polyketides and lipopeptides [69].

The balance between ROS production, immune activation, microbiota proliferation and bacterial profile changes along the digestive tract must be critical for proper establishment (replication and differentiation) of the *T*. *cruzi* parasite in the bug, with obvious consequences in terms of triatomine vectorial competence.

Early studies on triatomine endosymbionts described *R*. *rhodnii* (Actinobacteria) as responsible for providing nutrients (e.g. vitamins) that enable the successful growth of *R*. *prolixus* [37,70]. Several genes for the biosynthesis of natural products have been identified in the genome of *R*. *rhodnii* such as polyketide and fatty acid synthases, nonribosomal peptide synthases, phytoene, carotenoid and vitamin B synthases [71]. The genome of the Actinobacteria *Wigglesworthia*, a tsetse fly obligate symbiont, has genes related to the biosynthesis of chorismic and folic acids and phenylalanine [72], which may affect host physiology and vector competence to trypanosomes [73]. The Actinobacteria also produce a wide variety of secondary metabolites and antimicrobial compounds (antibacterial and antifungal) that may protect hosts against pathogens [53]. This might explain why Actinobacteria are dominant in the *T*. *sordida*’s anterior midgut.

## Conclusions

We have described the gut microbiota of field-collected *T*. *sordida* through all the bugs’ development stages and across the three major intestinal segments. Species in 12 genera were consistently found in all development stages and can be regarded as *T*. *sordida*’s ‘bacterial core set’. Some of these bacteria species, if proven cultivable, and non-pathogenic for humans or domestic animals, could be tested further for genetic tractability, stability after insertion, and fitness compared with wild type populations. They would hence become good candidates to be used in novel control strategies that make use of the vectors’ own microbiota to reduce pathogen transmission. For example, some bacteria can naturally control parasite loads through superactivation of the insect immune system, secretion of anti-pathogenic molecules, or by physically inhibiting their development inside the vector [23]. A second strategy is paratransgenesis, whereby specific bacteria are genetically transformed so that they secrete pathogen-killing molecules inside the vector [74,75] or synthesize double-stranded RNA molecules that interfere with the vectors’ development, survival, or reproduction [20]. The development of insecticide resistance has brought to our attention the immediate need we have to diversify our tools to control vectors and vector-borne diseases.

## Supporting information

S1 Text All supporting information. (PDF)
