# Supplementary material for: Field-collected Triatoma sordida from central Brazil display high microbiota diversity that varies with regard to developmental stage and intestinal segmentation
Source: PLoS Negl Trop Dis. 2018 Aug 23;12(8):e0006709. doi: 10.1371/journal.pntd.0006709 (PMC6138416; doi:10.1371/journal.pntd.0006709)
Supplement: S1 Text — (PDF) [file pntd.0006709.s001.pdf]

Field-collected *Triatoma sordida* from central Brazil display high microbiota diversity that varies with regard to developmental stage and intestinal segmentation

Short title: ***Triatoma sordida* intestinal microbiota**

Joana L. Oliveira[1,4], Juliano C. Cury[2], Rodrigo Gurgel-Gonçalves[3], Ana C. Bahia[4¶*], Fernando A. Monteiro[1,5¶*]

S1 Table. Analysis of similarity (ANOSIM) of the bacterial community of *T. sordida* intestine. Corrected P-values (<0.01) were calculated using the Bonferroni method. Significant comparisons are shown in italic. Significant and meaningful comparisons are shown both in italic and bold. Samples analyzed: entire intestine of 1st stage nymphs (1I); entire intestine of 2nd stage nymphs (2I); entire intestine of 3rd stage nymphs (3I); anterior midgut of 4th stage nymphs (4AM); posterior midgut of 4th stage nymphs (4PM); hindgut of 4th stage nymphs (4H); anterior midgut of 5th stage nymphs (5AM); posterior midgut of 5th stage nymphs (5PM); hindgut of 5th stage nymphs (5H); anterior midgut of females (FAM); posterior midgut of females (FPM); hindgut of females (FH); anterior midgut of males (MAM); posterior midgut of males (MPM); hindgut of males (MH).

	**1I**	**2I**	**3I**	**4AM**	**4PM**	**4H**	**5AM**	**5PM**	**5H**	**FAM**	**FPM**	**FH**	**MAM**	**MPM**	**MH**
**1I**	-	0.0682	0.0127	**0.0042**	**0.0040**	**0.0099**	**0.0011**	**0.0011**	**0.0011**	0.0504	0.0509	**0.0009**	0.0505	0.0505	**0.0007**
**2I**		-	0.0122	**0.0013**	**0.0053**	**0.0011**	0.0405	**0.0051**	**0.0045**	**0.0042**	**0.0051**	**0.0008**	0.0452	**0.0050**	**0.0008**
**3I**			-	**0.0011**	0.0100	**0.0008**	0.0421	0.0102	**0.0045**	0.0412	**0.0040**	**0.0008**	**0.0010**	0.0110	**0.0009**
**4AM**				-	**0.0001**	**0.0052**	0.0826	**0.0008**	**0.0051**	0.0155	**0.0015**	**0.0023**	0.0457	**0.0013**	**0.0009**
**4PM**					-	0.0008	0.3495	0.2010	**0.0012**	**0.0011**	0.0729	0.2733	0.0827	0.0802	**0.0022**
**4H**						-	**0.0094**	0.0280	0.0495	**0.0007**	0.2395	**0.0010**	**0.0008**	0.0999	**0.0098**
**5AM**							-	0.0290	**0.0039**	0.0222	**0.0052**	**0.0010**	**0.0012**	0.0119	**0.0010**
**5PM**								-	**0.0013**	0.0224	0.0829	**0.0042**	0.0822	0.0830	0.5115
**5H**									-	**0.0053**	0.3341	0.0110	0.1000	0.2113	0.0612
**FAM**										-	**0.0015**	0.0110	**0.0015**	**0.0010**	0.1002
**FPM**											-	**0.0083**	0.0322	0.0210	**0.0011**
**FH**												-	0.0112	0.3924	0.1001
**MAM**													-	0.0100	0.1021
**MPM**														-	**0.0010**
**MH**															-

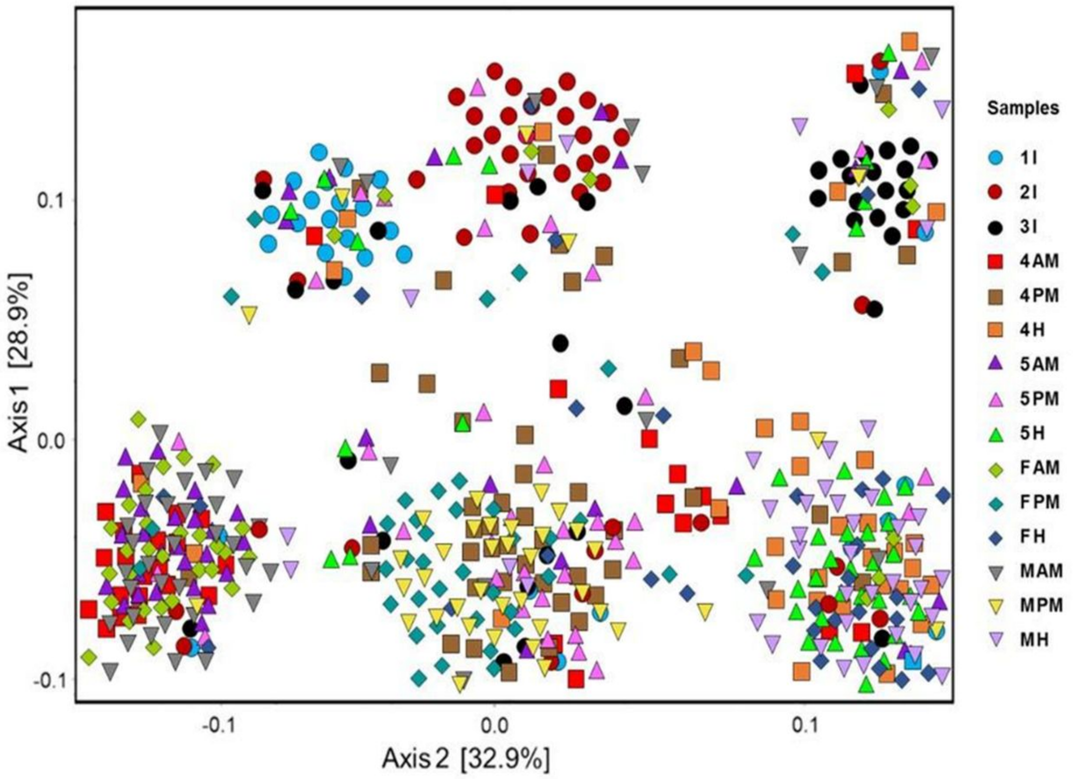

S1 Appendix. Principal coordinate analysis (PCoA) of weighted-UniFrac distances comparing the bacterial communities of *T. sordida* intestine. Weighted-UniFrac distances matrices were calculated for each sample using one representative sequence for each OTU (3%). Axes represent the greatest proportion of variances in the communities for each analysis. Samples analyzed: entire intestine of 1st stage nymphs (1I); entire intestine of 2nd stage nymphs (2I); entire intestine of 3rd stage nymphs (3I); anterior midgut of 4th stage nymphs (4AM); posterior midgut of 4th stage nymphs (4PM); hindgut of 4th stage nymphs (4H); anterior midgut of 5th stage nymphs (5AM); posterior midgut of 5th stage nymphs (5PM); hindgut of 5th stage nymphs (5H); anterior midgut of females (FAM); posterior midgut of females (FPM); hindgut of females (FH); anterior midgut of males (MAM); posterior midgut of males (MPM); hindgut of males (MH).

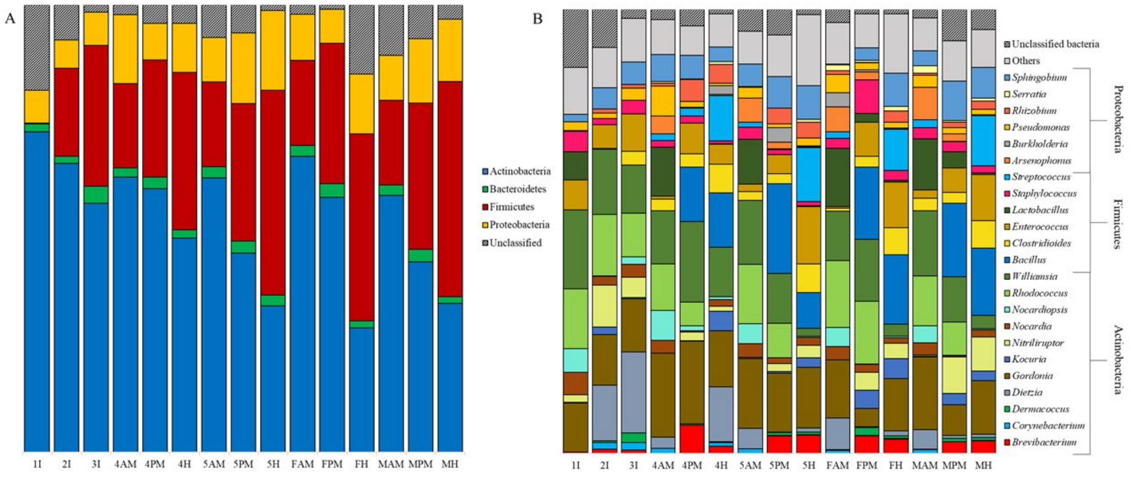

S2 Appendix. Relative abundance of bacterial phyla (A) and genera (B) found in *T. sordida* intestinal microbiota. Samples: entire intestine of 1st stage nymphs (1I); entire intestine of 2nd stage nymphs (2I); entire intestine of 3rd stage nymphs (3I); anterior midgut of 4th stage nymphs (4AM); posterior midgut of 4th stage nymphs (4PM); hindgut of 4th stage nymphs (4H); anterior midgut of 5th stage nymphs (5AM); posterior midgut of 5th stage nymphs (5PM); hindgut of 5th stage nymphs (5H); anterior midgut of females (FAM); posterior midgut of females (FPM); hindgut of females (FH); anterior midgut of males (MAM); posterior midgut of males (MPM); hindgut of males (MH).

S2 Table. Field expedition data. Number of collected specimens of *T. sordida* in three expeditions and positivity to *T. cruzi*.

Specimens collected	Field expeditions data				
	December 2013	May 2014	November 2014	Total	*T. cruzi* - infected
1st instar	2	0	5	7	0
2nd instar	8	2	17	27	0
3rd instar	5	2	9	16	0
4th instar	3	14	5	22	0
5th instar	7	26	35	68	0
Females	20	12	28	60	0
Males	32	35	37	104	0
Total	77	91	136	304	0

S3 Table. Coverage indices of *T. sordida* intestinal samples. Good's coverage of 454 pyrosequencing for each sample ($p < 0.05$) calculated with the Mothur program. Samples analyzed: entire intestine of 1st stage nymphs (1I); entire intestine of 2nd stage nymphs (2I); entire intestine of 3rd stage nymphs (3I); anterior midgut of 4th stage nymphs (4AM); posterior midgut of 4th stage nymphs (4PM); hindgut of 4th stage nymphs (4H); anterior midgut of 5th stage nymphs (5AM); posterior midgut of 5th stage nymphs (5PM); hindgut of 5th stage nymphs (5H); anterior midgut of females (FAM); posterior midgut of females (FPM); hindgut of females (FH); anterior midgut of males (MAM); posterior midgut of males (MPM); hindgut of males (MH).

**Samples**	**Good's coverage (%)**
**1I**	93.76
**2I**	96.13
**3I**	96.84
**4AM**	97.02
**4PM**	97.14
**4H**	96.03
**5AM**	97.20
**5PM**	97.20
**5H**	97.32
**FAM**	96.44
**FPM**	95.49
**FH**	97.23
**MAM**	97.35
**MPM**	97.16
**MH**	97.41

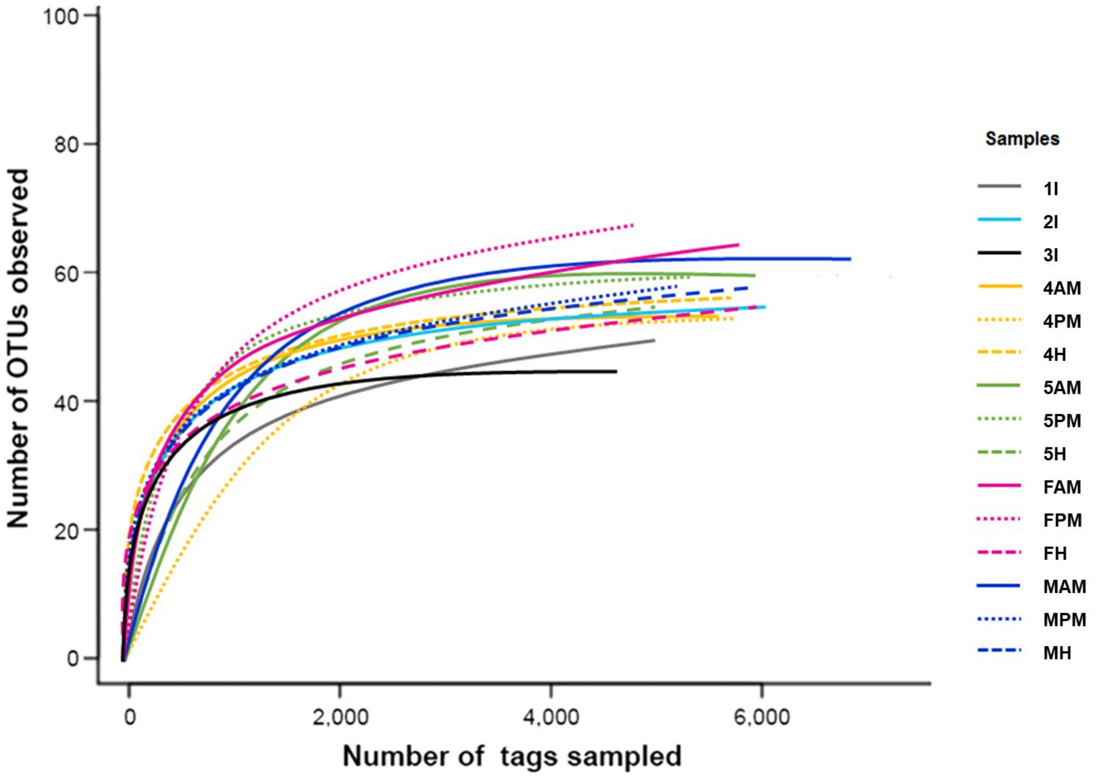

S3 Appendix. Rarefaction curves of 16S rRNA sequences from intestinal segments of *T. sordida* samples. Samples analyzed: entire intestine of 1st stage nymphs (1I); entire intestine of 2nd stage nymphs (2I); entire intestine of 3rd stage nymphs (3I); anterior midgut of 4th stage nymphs (4AM); posterior midgut of 4th stage nymphs (4PM); hindgut of 4th stage nymphs (4H); anterior midgut of 5th stage nymphs (5AM); posterior midgut of 5th stage nymphs (5PM); hindgut of 5th stage nymphs (5H); anterior midgut of females (FAM); posterior midgut of females (FPM); hindgut of females (FH); anterior midgut of males (MAM); posterior midgut of males (MPM); hindgut of males (MH).

S4 Table. Richness and diversity indices for *T. sordida* intestinal microbiota in different intestinal segments. OTUs were defined based on 97% identity cutoff. Samples analyzed: Anterior midgut (AM = 4_AM+5_AM+F_AM+M_AM); Posterior midgut (PM = 4_PM+5_PM+F_AM+M_AM); Hindgut (H = 4_H+5H+F_H+M_H).

Samples	Nº of observed OTUs	Chao1	Shannon
**AM**	46	47.5 ± 0.70	3.50 ± 0.75
**PM**	35	36.5 ± 0.50	3.25 ± 0.25
**H**	33	38.5 ± 1.50	2.30 ± 0.30

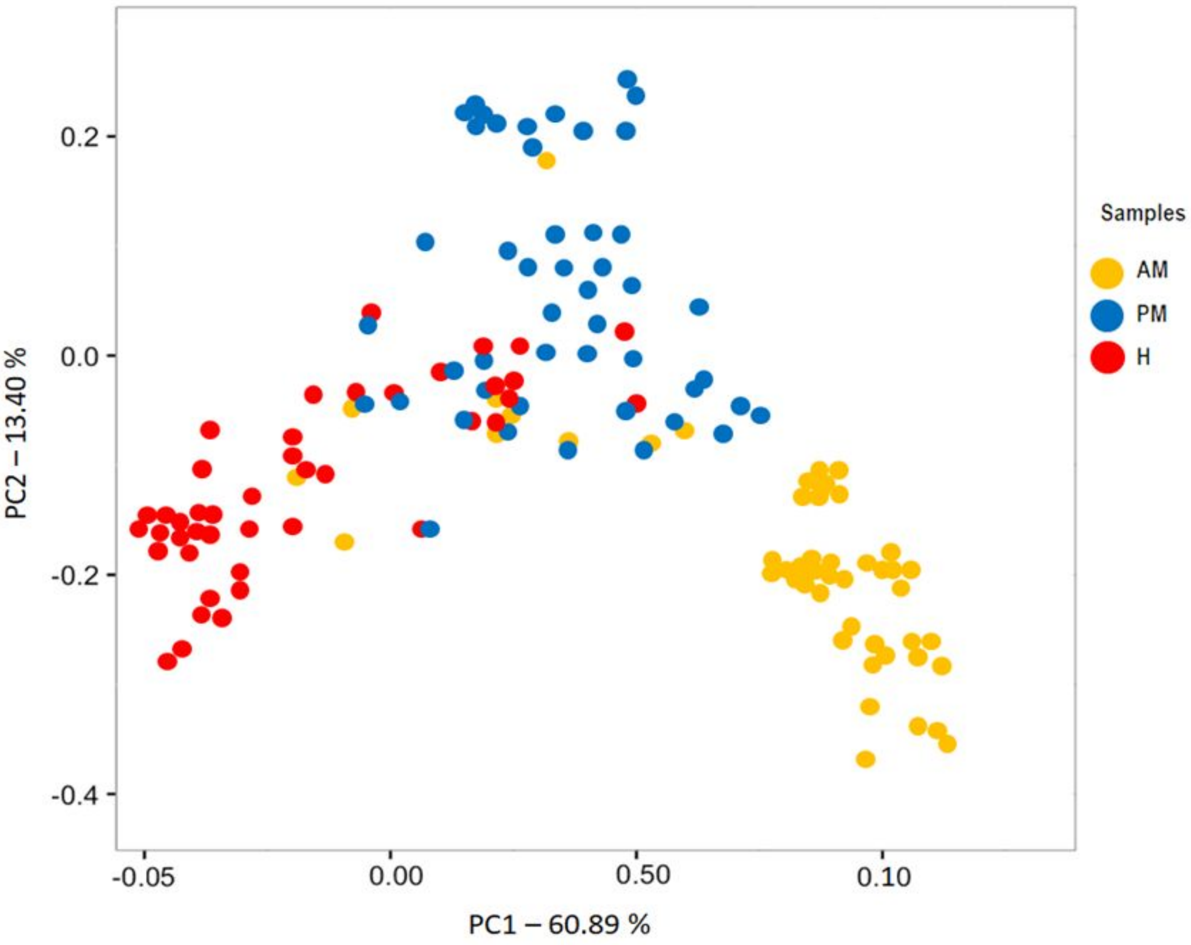

S4 Appendix. Principal coordinate analysis (PCoA) of weighted-UniFrac distances comparing the bacterial communities of *T. sordida* intestinal segments. Weighted-UniFrac distances matrices were calculated for each sample using one representative sequence for each OTU (3%). Axes represent the greatest proportion of variances in the communities for each analysis. Samples analyzed: Anterior midgut (AM = 4_AM+5_AM+F_AM+M_AM); Posterior midgut (PM = 4_PM+5_PM+F_AM+M_AM); Hindgut (H = 4_H+5H+F_H+M_H).

S5 Table. Analysis of similarity (ANOSIM) of the bacterial community of intestinal segments of *T. sordida*. Corrected P-values (<0.025) were calculated using the Bonferroni method. Significant comparisons are shown in bold. Samples analyzed: Anterior midgut (AM = 4_AM+5_AM+F_AM+M_AM); Posterior midgut (PM = 4_PM+5_PM+F_AM+M_AM); Hindgut (H = 4_H+5H+F_H+M_H).

	AM	PM	H
**AM**	-	0.0357	**0.0086**
**PM**		-	**0.0054**
**H**			-

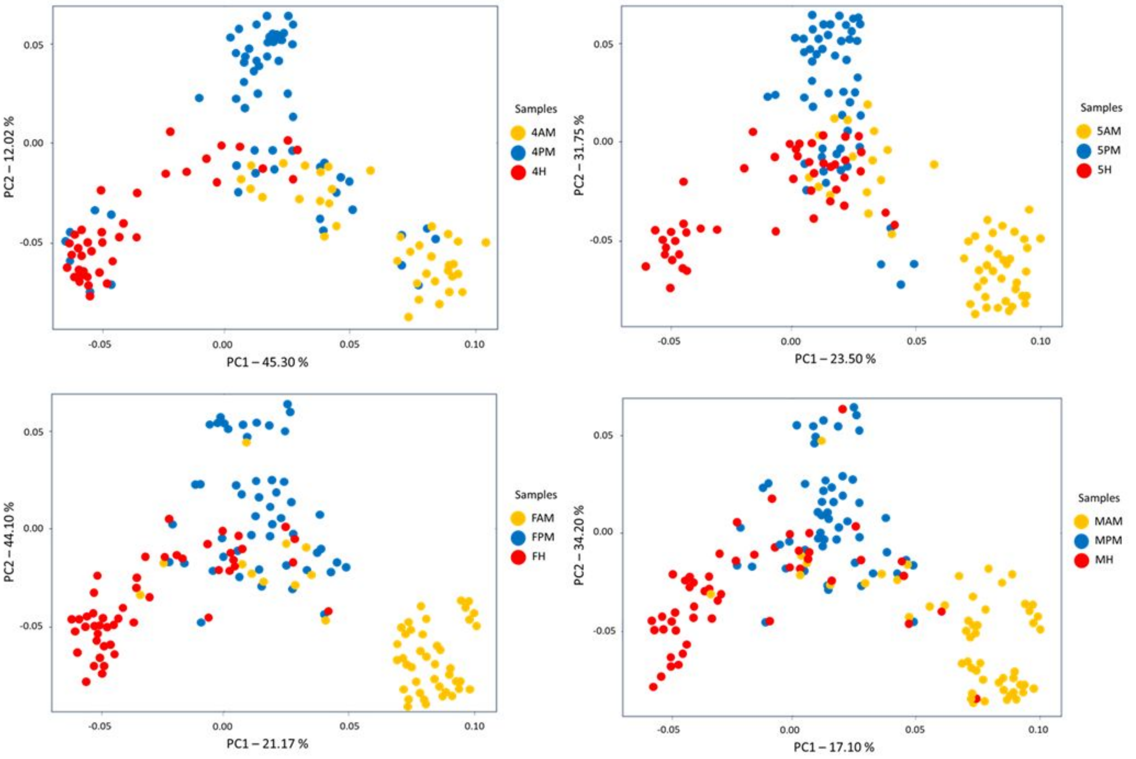

S5 Appendix. Principal coordinate analysis (PCoA) of weighted-UniFrac distances comparing the bacterial communities of *T. sordida* intestine in different intestinal segments. Weighted-UniFrac distances matrices were calculated for each sample using one representative sequence for each UTO (3%). Axes represent the greatest proportion of variances in the communities for each analysis. Samples analyzed:Anterior midgut of 4th stage nymphs (4AM); posterior midgut of 4th stage nymphs (4PM); hindgut of 4th stage nymphs (4H); anterior midgut of 5th stage nymphs (5AM); posterior midgut of 5th stage nymphs (5PM); hindgut of 5th stage nymphs (5H); anterior midgut of females (FAM); posterior midgut of females (FPM); hindgut of females (FH); anterior midgut of males (MAM); posterior midgut of males (MPM); hindgut of males (MH).

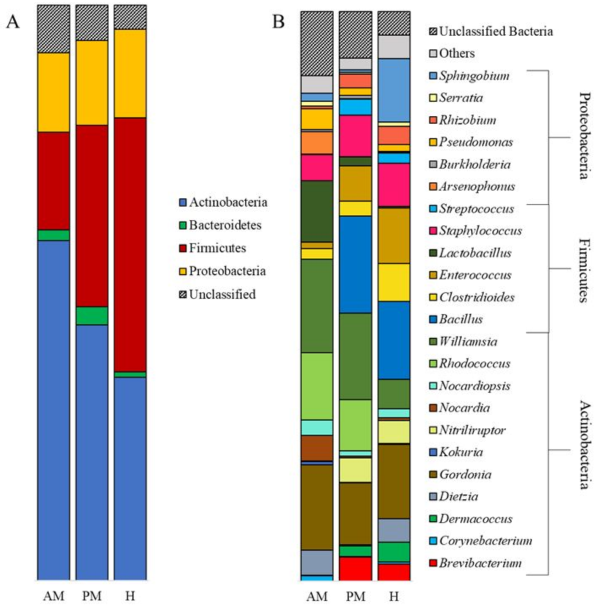

S6 Appendix. Relative abundance of bacterial phyla (A) and genera (B) found in *T. sordida* intestinal microbiota. Samples: **a**nterior midgut of 4th instar, 5th instar, female and male (AM); posterior midgut of of 4th instar, 5th instar, female and male and hindgut of 4th instar, 5th instar, female and male (H). I = entire intestine. AM = anterior midgut, PM = posterior midgut, H = hindgut.
